# Simultaneous in vivo imaging with PET and SPECT tracers using a Compton-PET hybrid camera

**DOI:** 10.1038/s41598-021-97302-7

**Published:** 2021-09-09

**Authors:** Mizuki Uenomachi, Miwako Takahashi, Kenji Shimazoe, Hiroyuki Takahashi, Kei Kamada, Tadashi Orita, Kenichiro Ogane, Atsushi B. Tsuji

**Affiliations:** 1 Department of Nuclear Engineering and Management, School of Engineering, The University of Tokyo, 7-3-1, Hongo, Bunkyo-ku, Tokyo, Japan; 2 National Institute of Radiological Sciences, National Institutes for Quantum and Radiological Science and Technology, 4-9-1, Inage, Chiba, Chiba Japan; 3 Department of Bioengineering, School of Engineering, The University of Tokyo, 7-3-1, Hongo, Bunkyo-ku, Tokyo, Japan; 4 JST, PRESTO, Saitama, 332-0012 Japan; 5 Institute of Engineering Innovation, School of Engineering, The University of Tokyo, 2-11-16, Yayoi, Bunkyo-ku, Tokyo, Japan; 6 Tohoku University, 2-1-1, Katahira, Sendai, Miyagi Japan; 7 Kavli Institute for the Physics and Mathematics of the Universe (WPI), The University of Tokyo, Kashiwa, Chiba Japan; 8 Department of Surgery, Graduate School of Medicine, The University of Tokyo, 7-3-1, Hongo, Bunkyo-ku, Tokyo, Japan; 9 Department of Nuclear Medicine, International University of Health and Welfare, 1-4-3, Minato-ku, Tokyo, Japan

**Keywords:** Biomedical engineering, Molecular imaging

## Abstract

Positron-emission tomography (PET) and single-photon-emission computed tomography (SPECT) are well-established nuclear-medicine imaging methods used in modern medical diagnoses. Combining PET with ^18^F-fluorodeoxyglucose (FDG) and SPECT with an ^111^In-labelled ligand provides clinicians with information about the aggressiveness and specific types of tumors. However, it is difficult to integrate a SPECT system with a PET system because SPECT requires a collimator. Herein, we describe a novel method that provides simultaneous imaging with PET and SPECT nuclides by combining PET imaging and Compton imaging. The latter is an imaging method that utilizes Compton scattering to visualize gamma rays over a wide range of energies without requiring a collimator. Using Compton imaging with SPECT nuclides, instead of the conventional SPECT imaging method, enables PET imaging and Compton imaging to be performed with one system. In this research, we have demonstrated simultaneous in vivo imaging of a tumor-bearing mouse injected with ^18^F-FDG and an ^111^In-antibody by using a prototype Compton-PET hybrid camera. We have succeeded in visualizing accumulations of ^18^F-FDG and ^111^In-antibody by performing PET imaging and Compton imaging simultaneously. As simultaneous imaging utilizes the same coordinate axes, it is expected to improve the accuracy of diagnoses.

## Introduction

Since the beginning of research on nuclear-medicine imaging technology in the 1950s, single-photon-emission computed tomography (SPECT)^1^ and positron-emission tomography (PET)^2,3^ have become important imaging methods in both medical research and diagnosis. SPECT uses a collimator to define the direction of incoming gamma rays. Therefore, low-energy gamma rays (50–400 keV) are used for SPECT. Conversely, PET uses positron-emitting nuclides and detects the coincidence between two 511 keV gamma rays emitted in opposite directions when a positron annihilates with an electron. Therefore, PET imaging can visualize only a PET tracer. However, since PET does not require a collimator, its sensitivity is higher than that of SPECT. PET with ^18^F-fluorodeoxyglucose (FDG) is most widely used to detect malignant tumors via imaging glucose metabolism, which is accelerated with more aggressive tumors^4,5^. However, glucose metabolic imaging is less specific to individual types of tumors. Tumor-specific imaging is now available using SPECT with ^111^In-labeled ligands, such as the anti-CD20 antibody that targets CD20 positive lymphoma, or pentetreotide that targets somatostatin receptors^6,7^. The combination of PET with ^18^F-FDG and SPECT with an ^111^In-labelled ligand is helpful both in guiding the method of treatment as well as in diagnosis. However, since the PET and SPECT modalities require separate devices and the resolutions are considerably different, the matching of lesion positions is based on experience. As simultaneous imaging with PET and SPECT nuclides yields images on the same coordinate axes, the accuracy of the diagnosis can be significantly improved by combining these techniques.

**Figure 1:**
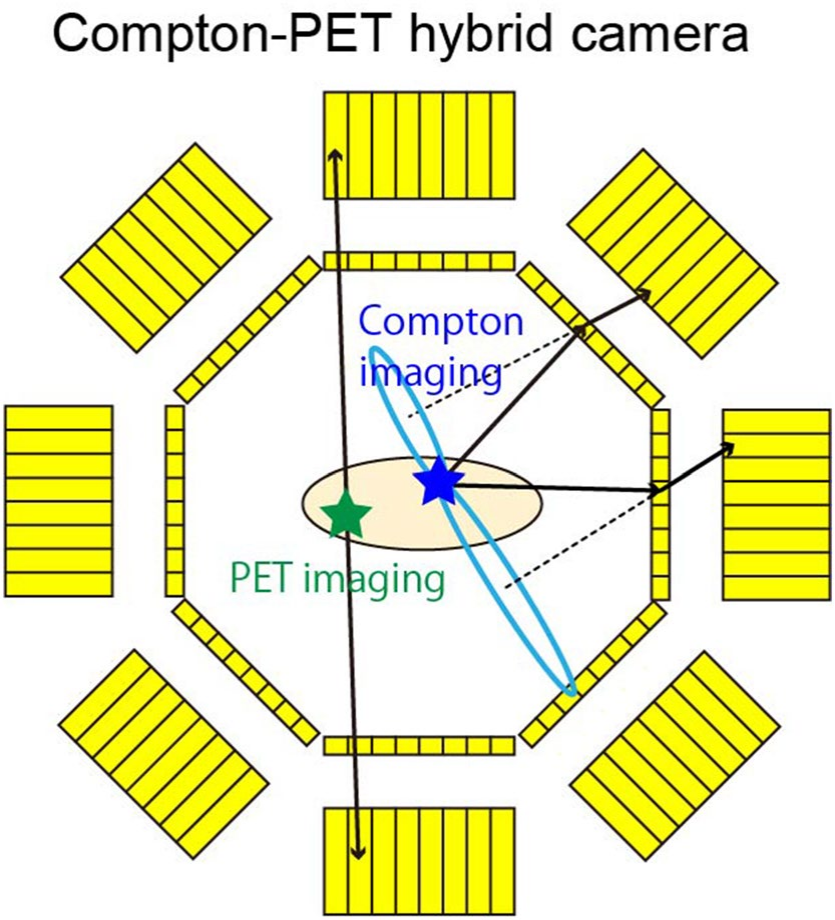
Concept of the Compton-PET hybrid camera. The PET tracers are visualized using coincidence detection of annihilation gamma rays through opposing absorbers. The SPECT tracers are visualized using coincidence detection of a Compton-scattered gamma ray (in a scatterer) and a fully absorbed gamma ray (in an absorber). Green and blue stars represent PET and SPECT tracers, respectively. The solid black lines represent two gamma rays, here scattered by separate scatterers (thin yellow boxes) before being absorbed in separate absorbers (thick yellow boxes). The dashed lines represent the axes of the two Compton cones, and the blue ovals represent their bases. The figure was created using the software (Adobe Illustrator, Illustrator CC 24, https://www.adobe.com/products/illustrator.html).

Although the technology to enable simultaneous imaging with PET and SPECT nuclides is required in nuclear medicine, it is difficult to integrate a SPECT system with a PET system because SPECT requires a collimator. Studies of multi-tracer imaging have been conducted by using the conventional SPECT imaging method^8–11^. Typically, multi-tracer SPECT imaging is performed for nuclides that emit low-energy gamma rays^8–10^. A SPECT system that can visualize both PET tracers and SPECT tracers has also been developed^11^. However, since this system uses collimators in detecting the 511 keV gamma-rays originating from a PET tracer as single photons, the sensitivity of the PET tracer is reduced compared with that obtained by PET imaging^11^. Recently, Compton imaging^12^ based on Compton scattering has been studied for applications in the fields of space^13,14^, nuclear^15–17^, and medical research^18–21^. Compton imaging has the capability for multi-nuclide imaging without requiring a collimator. Some groups have reported in vivo multi-tracer imaging by using Compton imaging^18–21^. In particular, references 10 and 11 reported simultaneous in vivo Compton imaging with a PET tracer and a SPECT tracer using a Si/CdTe Compton camera. However, simultaneous in vivo multi-tracer imaging that employs the principles of both PET imaging and Compton imaging has not yet been reported, although PET imaging can visualize a PET tracer with higher sensitivity and spatial resolution than those of Compton imaging. We have recently proposed a new simultaneous multi-nuclide imaging technology that combines PET imaging and Compton imaging^22^. Figure 1 shows the concept of the Compton-PET hybrid camera. It uses conventional PET imaging with coincidence detection of annihilation gamma rays to visualize the PET nuclides. The SPECT nuclides are visualized using Compton imaging. By detecting the coincidence between a Compton-scattered gamma ray of energy $$E_{s} $$ and a fully absorbed gamma ray of energy $$E_{a}$$, the angle of incidence $$\theta$$ of the gamma rays can be obtained via the following equation:

1 $$ \begin{array}{*{20}c} {\cos \theta = 1 + m_{0} c^{2} \left( {\frac{1}{{E_{s} + E_{a} }} - \frac{1}{{E_{a} }}} \right),} \\ \end{array} $$

where $m_{0}$ is the electron rest mass and $c$ is the speed of light. Hence, the location of a source can be restricted to a conical surface (the Compton cone) from information about the energies and positions of the Compton-scattered gamma rays and the fully absorbed gamma rays. In this system, coincidence events between 511 keV gamma rays from two scatterer–absorber modules are regarded as PET events, whereas coincidence events between a scatterer and an absorber module are regarded as Compton events. The PET events are used for PET imaging by overlaying lines of response (LORs) to visualize the PET tracers, and Compton events are used for Compton imaging by overlaying Compton cones to visualize the SPECT tracers. In a previous report^22^, we demonstrated simultaneous imaging with ^18^F (PET nuclide) and ^111^In (SPECT nuclide) for proof of principle with a prototype of Compton-PET hybrid camera, which was optimized for low energy gamma-ray (150–400 keV) Compton imaging and PET imaging. In this study, we show the feasibility of such a Compton-PET hybrid camera through simultaneous in vivo imaging with PET and SPECT tracers of a tumor-bearing mouse injected with ^18^F-FDG (PET tracer) and an ^111^In-labeled ligand (SPECT tracer).

## Results

### Basic performance

**Figure 2:**
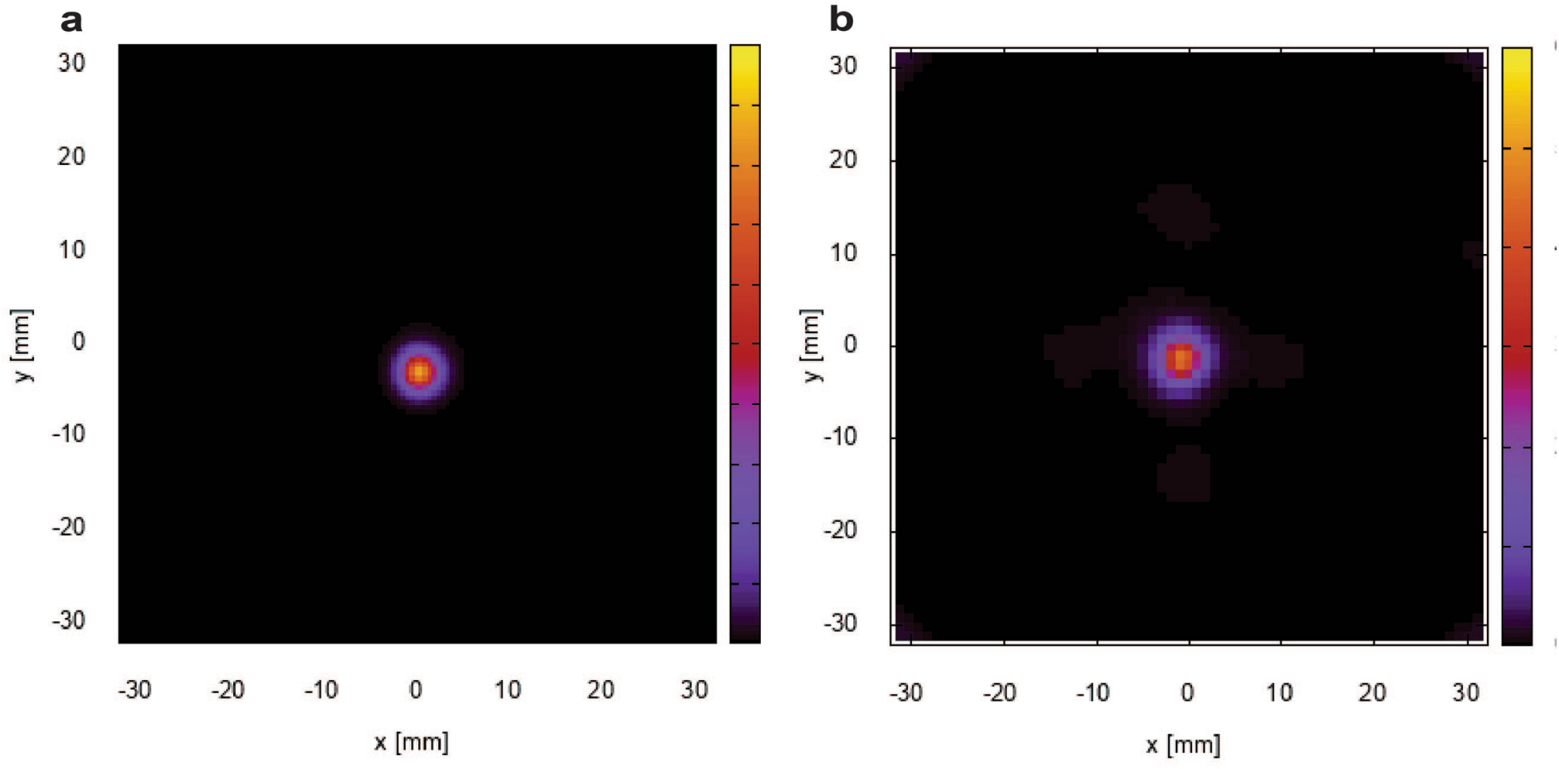
Imaging results for ^22^Na and ^18^F point sources. **(a**) PET image of a ^22^Na point source obtained using the BP method. (**b**) Compton image of an ^18^F point sources obtained with the MLEM method at a distance of 30 mm.

We evaluated the energy resolution, the angular resolution measure (ARM), the spatial resolution, and the time resolution of the prototype system. The detector configuration to evaluate ARM and spatial resolution is described in the Method section. The energy resolution of scatterers was approximately 18.2% (full width at half maximum: FWHM) at 59.54 keV. The energy resolution of gamma rays above 100 keV was approximately 11–15% (FWHM) and was limited by the voltage range of the ADCs. The ARM is commonly used as a parameter to represent the angular resolution of Compton camera. We calculated it from the difference between the angle calculated from deposited energies and the angle from interaction positions. The ARM includes uncertainties caused by the position resolution, energy resolution and Doppler broadening effect. The angular resolutions at 245 keV and 511 keV using point sources of ^111^In and ^18^F were 17.2° (FWHM) and 11.6° (FWHM), respectively. The calculated ARMs were considered in the Compton imaging reconstruction. Figure 2a shows the reconstructed PET image of a ^22^Na point source at a distance of 30 mm by using the back-projection (BP) method. We evaluated the spatial resolution by measuring the ^22^Na point source and evaluating a point-spread-function of the reconstructed image. The spatial resolutions of PET imaging at 511 keV along the horizontal and vertical axes were 3.3 mm (FWHM) and 3.3 mm (FWHM), respectively. Figure 2b shows the reconstructed Compton image of an ^18^F point source at a distance of 30 mm by using the maximum-likelihood expectation–maximization (MLEM) method^23^ after 60 iterations. The spatial resolutions of Compton imaging at 511 keV along the horizontal and vertical axes were 4.2 mm (FWHM) and 3.8 mm (FWHM), respectively. The time resolution was 47.7 ns and was mostly restricted by the slew rate of the CMOS amplifiers on the signal-processing boards.

### Simultaneous imaging of PET and SPECT nuclides with micro tubes

**Figure 3:**
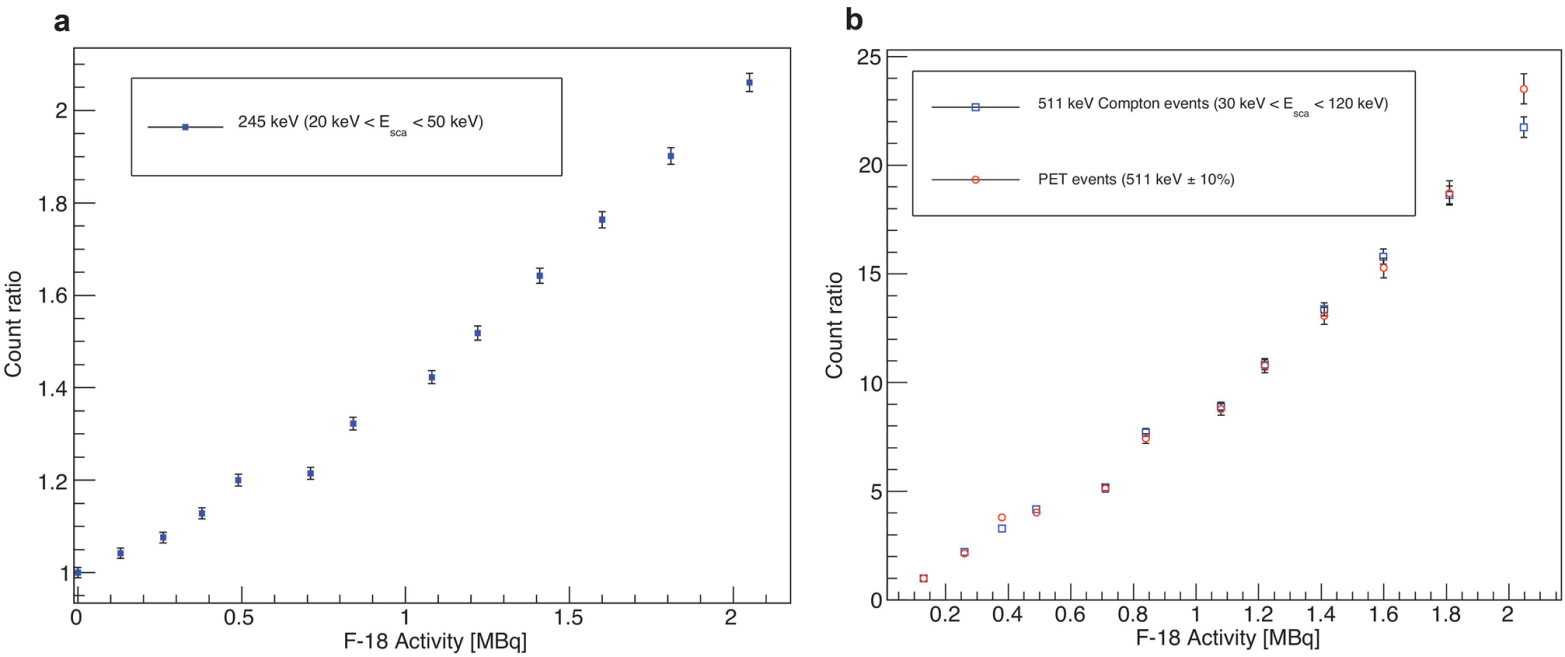
Relation between Compton events and ^18^F radioactivity. **(a**) Relation between the 245 keV Compton events used for Compton imaging and ^18^F radioactivity. The *y*-axis represents the ratio of ^111^In Compton events measured with ^18^F to Compton events measured with no ^18^F source. As the ^18^F radioactivity increases, Compton events due to 245 keV gamma rays also increase. (**b**) Relation between Compton events (PET events) at 511 keV used for Compton imaging (PET imaging) and ^18^F radioactivity. The *y*-axis is the ratio of Compton events (PET events) measured with various ^18^F sources to Compton events measured with a 0.13 MBq ^18^F source.

We performed simultaneous ^18^F-FDG (PET nuclide) and ^111^In (SPECT nuclide) imaging with microtubes before conducting in vivo imaging. The detail of imaging system and experimental setup are described in the Method section. The nuclide ^18^F is a positron emitter, which yields a pair of annihilation gamma rays with an energy of 511 keV. The nuclide ^111^In emits gamma rays with energies of 171 keV and 245 keV. As the gamma-ray energies from the SPECT nuclides are lower than 511 keV—the gamma-ray energy of the PET nuclides—crosstalk is caused by the scattered 511 keV gamma rays, and this affects Compton-image reconstruction using low-energy gamma rays^24^. We measured the count ratio of ^18^F-FDG to ^111^In using various combinations of each radioactivity. Figure 3a shows the count ratio calculated by dividing ^111^In Compton events measured with ^18^F by that measured with no ^18^F source and Fig. 3b shows the count ratio calculated by dividing ^18^F Compton events or PET events by those of a 0.13 MBq ^18^F source. For all the measurements, the radioactivity of ^111^In was nearly constant (~ 1 MBq); however, as the radioactivity of ^18^F increased, so did the Compton events involving 245 keV gamma rays from ^111^In, which was used for image reconstruction. This indicates that the effect of crosstalk, which cannot be distinguished by the energy and time windows, increases with the ^18^F radioactivity. Conversely, we assume that the 511 keV Compton events have few crosstalk elements, because the rate of increase in the 511 keV Compton events is approximately the same as that of the PET events.

**Figure 4:**
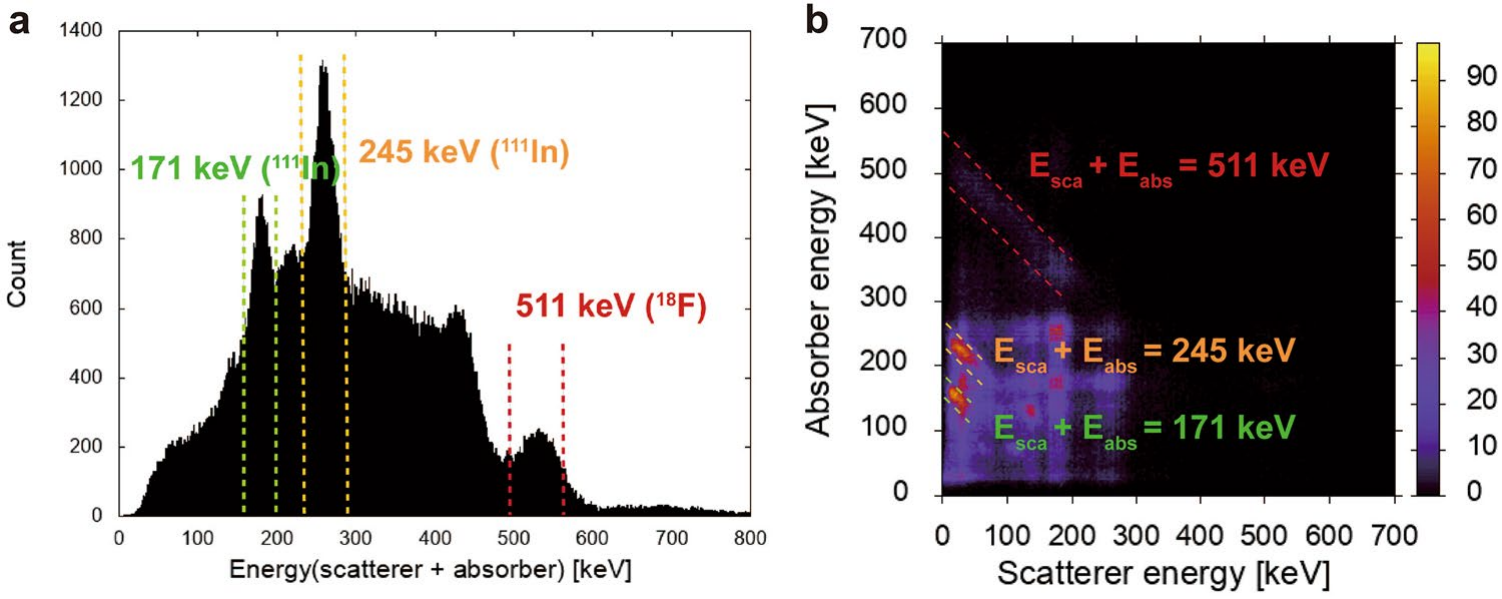
Results for Compton events. (**a**) The spectrum of energies deposited in the scatterer and absorber by Compton events. (**b**) A 2D map of the scattered energy and the absorbed energy of Compton events. The area between the red dotted lines corresponds to Compton events due to 511 keV gamma rays from ^18^F-FDG. The areas between the green and orange dotted lines correspond to Compton events produced by the 171 keV and 245 keV gamma rays from ^111^In.

**Figure 5:**
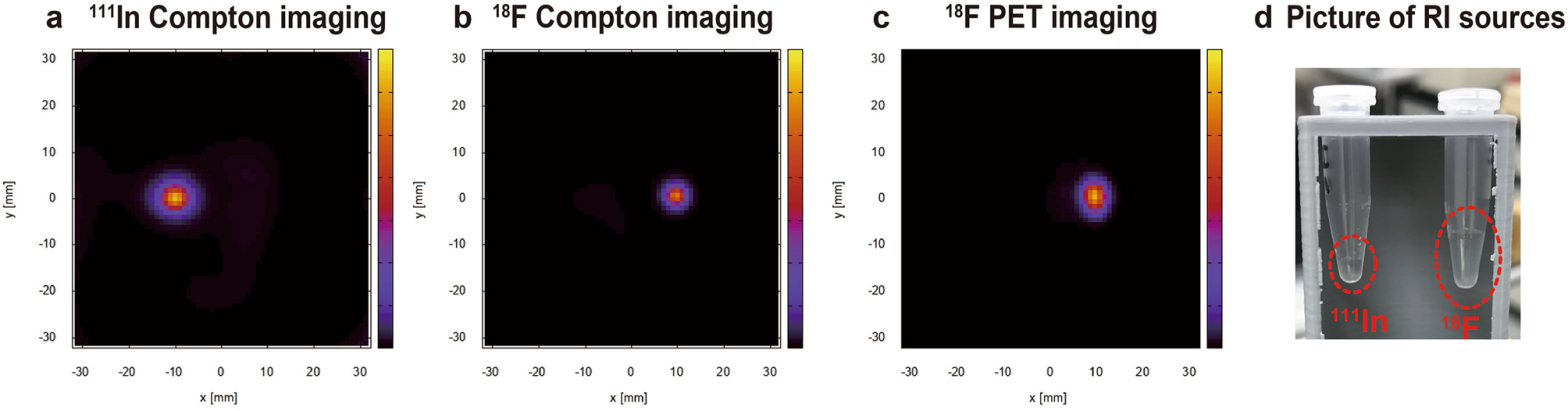
Simultaneous ^111^In and ^18^F imaging results. (**a**) A reconstructed image of an ^111^In radioisotope obtained by Compton imaging, using 245 keV gamma rays for the reconstruction. (**b**) A reconstructed image of an ^18^F radioisotope obtained by Compton imaging, using 511 keV gamma rays for the reconstruction. (**c**) A reconstructed image of the ^18^F radioisotope obtained by PET imaging. (**d**) A picture of RI sources in micro tubes.

Figures 4 and 5 show the results from a simultaneous ^18^F and ^111^In imaging experiment with radioactivities of 0.2 MBq and 1.0 MBq, respectively. Figure 4a shows the spectrum of energies deposited in the scatterer and absorber from Compton events. The peak in the area between the red dotted lines corresponds to Compton events due to the 511 keV gamma rays from ^18^F-FDG. The peaks in the areas between the green and orange dotted lines correspond to Compton events produced by the 171 keV and 245 keV gamma rays from ^111^In. The peak around 420 keV indicates the chance coincidence between 171 and 245 keV photoelectric absorption events. A 2D map of the scattered energy and the absorbed energy owing to Compton events is shown in Fig. 4b. Owing to the setup geometry of the scatterer and absorber, the scattered energy detected was limited. In particular, the energies deposited in the scatterer by low-energy 171 keV and 245 keV gamma rays were only 50 keV or less. For 245 keV gamma-ray imaging, Compton events with scattered energies of 50 keV or less were used to reduce the background for reconstruction. Besides, there are three hot spots in Fig. 4b, which were mostly derived from chance coincidences of ^111^In: between 171 and 245 keV photoelectric absorption events, between 171 keV photoelectric absorption events, between 245 keV Compton edge events (~ 125 keV). Figure 5 shows the reconstruction results for ^18^F-FDG and ^111^In obtained from PET imaging and Compton imaging. The Compton images were reconstructed by using the maximum-likelihood expectation–maximization (MLEM) method. The PET image was reconstructed using the back-projection (BP) method. Figure 5a, b show Compton imaging results obtained using the 245 keV gamma rays (from ^111^In) and 511 keV gamma rays (from ^18^F), and Fig. 5c shows the PET imaging results. The location of each source appears clearly in these images. Although ^18^F can be visualized with both Compton imaging and PET imaging, the shape of the ^18^F source was more clearly visualized in the PET image than in the Compton image.

### Simultaneous in vivo imaging with PET and SPECT tracers

**Figure 6:**
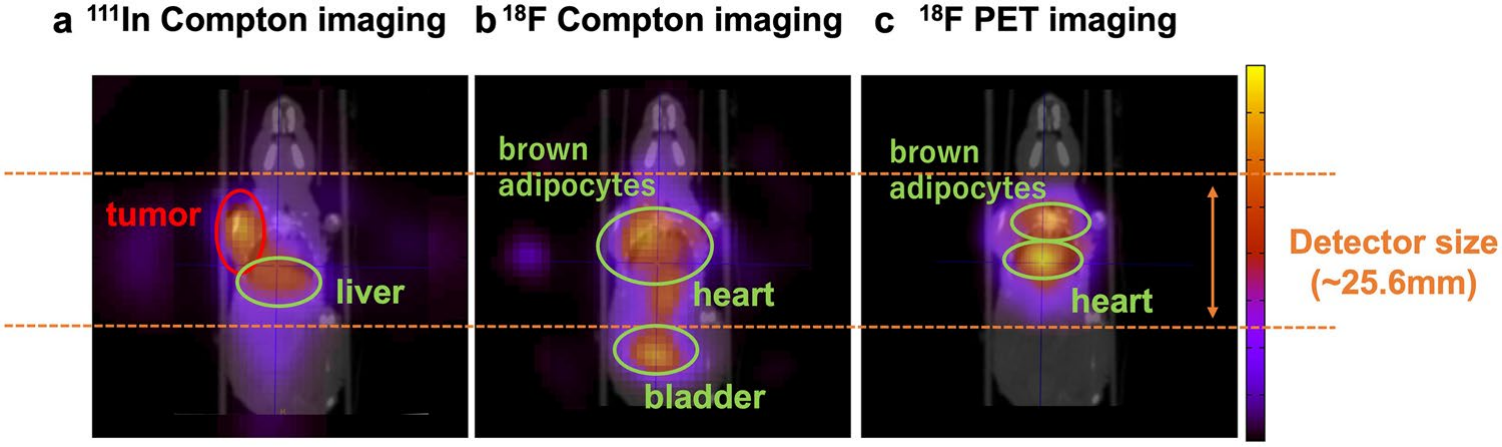
Simultaneous ^111^In and ^18^F in vivo imaging results. (**a**) A reconstructed image of the ^111^In antibody obtained by Compton imaging. The CT image and the reconstructed image are superimposed. The location of the tumor and the liver are visualized. (**b**) A reconstructed image of ^18^F-FDG obtained by Compton imaging. The location of the bladder is strongly visualized. (**c**) A reconstructed image of ^18^F-FDG obtained by PET imaging. The accumulation in the field of view (FOV), which is determined by the detector size, is visualized.

Next, we demonstrated simultaneous in vivo imaging with a male nude mouse bearing an SY tumor, a small-cell lung-cancer cell line. As the PET tracer and the SPECT tracer, we used ^18^F-FDG and an ^111^In-antibody (67A2; anti-c-kit mouse monoclonal antibody^25^), respectively. The mouse was placed on the stage in an upright position during the measurement. The imaging results are shown in Fig. 6. The Compton images and PET image were reconstructed by using the MLEM method and the BP method, respectively. We also took a CT scan of the mouse in a horizontal position. The tumor was on the left shoulder. The reconstructed images are superimposed on the CT image. Figure 6a is the reconstructed ^111^In image obtained by Compton imaging with 245 keV gamma rays. The ^111^In-antibody accumulated strongly in the tumor, and accumulation in the liver is also visualized. Figure 6b, c show the ^18^F-FDG images. The tracer ^18^F-FDG is visualized in several organs—for example, in the bladder, heart, and brown adipocytes. The reconstructed image obtained by Compton imaging with 511 keV gamma rays shows the ^18^F-FDG accumulation in the whole body. In Fig. 6b, the accumulation in the heart, brown adipocytes, and bladder is visualized. Conversely, PET imaging visualized the heart and brown adipocyte clearly; however, the bladder was not visualized because the PET field of view (FOV) is limited by the detector size (Fig. 6c). The accumulation of ^18^F-FDG in the tumor was not clearly visible in either of these images. A low uptake of ^18^F-FDG in an SY tumor was also reported in references^26,27^. After in vivo imaging, we also measured the radioactivity of some organs in the same mouse for an experiment and those in another mouse injected with only an ^111^In-antibody by using an auto-well gamma counter (Table 1). The amount of ^111^In anti-body injected into two mice was almost same (detailed in Method section), and these measurements were conducted approximately 3 days after the injection. For both mice the highest ^111^In radioactivity was located in the tumor, with a secondary accumulation in the liver. This result corresponds to the ^111^In Compton-imaging result. However, the radioactivity of ^18^F in organs was not accurately measured by the gamma counter because of a pile-up caused by the ^111^In radiation energies. This experiment was approved by the Committee of Care and Use of Laboratory Animals of National Institute of Radiological Sciences and was performed in compliance with the institutional guidelines.

**Table 1:** The ^111^In radioactivity of some organs and the tumors measured by an auto-well gamma counter after excision from tumor-bearing mice.

Organ	Mouse with ^18^F and ^111^In ^111^In (kBq/g)	Mouse with only ^111^In ^111^In (kBq/g)
Blood	67.4	71.3
Heart	16.8	17.8
Lung	36.6	38.7
Liver	74.6	78.9
Spleen	34.8	36.7
Kidney	19.8	21.0
Bone	9.6	10.2
Colon	9.0	9.5
Bladder	15.6	16.5
Tumor	174.1	184.0

## Discussion

Herein, we have demonstrated simultaneous in vivo imaging with PET and SPECT nuclides of a tumor-bearing mouse using our Compton-PET hybrid camera. The PET nuclides can be visualized using both Compton imaging and PET imaging; however, PET imaging can visualize the shape of ^18^F source more clearly than Compton imaging. The spatial resolution of the PET imaging obtained with our Compton-PET hybrid camera is not as good as that of commercial small-animal PET scanners^28,29^; however, this prototype Compton-PET hybrid camera is not sufficiently optimized for PET imaging. Consequently, there is room to improve the spatial resolution in terms of the detector pixel size—which mostly determines the spatial resolution—the time resolution, and the method of reconstruction. With respect to the system design, a ring system—which is the general design of PET—is ideal for quantitative imaging in the future. This also contributes to the improvement of Compton imaging as well as PET imaging. In general measurement from one angle by using one Compton camera, the spatial resolution in the direction vertical to the detector is inferior. Recently, its improvement by multi-angle measurement was reported^30^, and Tashima et al.^31^ succeeded to perform high quality in vivo Compton imaging by using the ring system. We are going to build a ring Compton-PET hybrid camera as the second prototype imaging system. Moreover, PET imaging is a well-established method, and the spatial resolution has been improved by using information about the depth of interaction (DOI)^32–34^ and the time-of-flight (TOF)^35–37^. These techniques also can be applied to our Compton-PET hybrid camera to improve the spatial and the time resolution.

In this research, the radioactivities of ^18^F and ^111^In for in vivo imaging were determined to be less affected by 511 keV scattered photons; however, this crosstalk was not corrected here. Although the Compton images of ^111^In-antibody and ^18^F-FDG visualized their accumulation, some artifacts appeared. In principle, energy information of Compton scattering gives us only the angle information of incoming gamma-ray unlike a PET event that determines the direction. There, Compton imaging is performed by drawing Compton cones, of which a part contributes to artifacts in the reconstructed image. Besides, crosstalk events caused by higher gamma-rays also contributes to artifacts in multi-tracer imaging. In in vivo imaging of larger animals or humans, the photons scattering within the body will contribute to additional crosstalk in the Compton events as well as backgrounds in PET events, therefore, the scatter correction is required. The scatter correction method for Compton imaging has not been studied so much yet, whereas that for PET imaging has been developed and widely used in the field of PET imaging. Recently, we demonstrated the scatter correction method in the Compton imaging system by setting arbitrary scattering points on the attenuating material^38^. As the method to reduce crosstalk artifacts, Sakai et al.^24^ demonstrated a dual-energy-window scatter correction, which is used in SPECT imaging, for multi-nuclide Compton imaging. Another candidate as crosstalk reduction method, especially for ^111^In, is the double photon coincidence method^39–42^. ^111^In emits successive gamma-rays of 171 keV and 245 keV via an intermediate state, therefore, the coincidence detection of these gamma-rays could result in the drastic crosstalk reduction. By using these techniques, the scattered photons and crosstalk events could be reduced, resulting in the improvement of Compton images.

Not only SPECT nuclides but also therapeutic nuclides can be visualized simultaneously with PET nuclides. For example, ^131^I—which mainly emits 364 keV gamma rays—is widely used for thyroid-cancer therapy^43^, and ^177^Lu—which mainly emits 113 keV and 208 keV gamma rays—is a promising therapeutic nuclide that is used for neuroendocrine-tumor therapy^44^. The energies of these gamma rays are suitable for low-energy gamma-ray Compton imaging; therefore, our Compton-PET hybrid camera can afford simultaneous imaging of therapeutic and diagnosis nuclides, thus eliminating the effects of organ movement. In addition to improving the accuracy of diagnosis and reducing the patients’ burden caused by several exams, the Compton-PET hybrid camera may become a useful modality for research into promising therapeutic nuclides.

## Methods

### Detector

The detector configuration of the prototype Compton-PET hybrid camera comprises two modules of scatterers and absorbers, each containing pixelated scintillator arrays and silicon photomultipliers (SiPMs). To visualize a SPECT tracer using Compton imaging requires suitable energy resolution. Therefore, we chose a high-resolution-type Ce:Gd_3_Al_2_Ga_3_O_12_ (HR-GAGG) scintillator^45^ for this study. A GAGG scintillator has the characteristics of desirable energy resolution (4% with an avalanche photodiode [APD]), high light yield (56,000 photons/MeV), high density (6.63 g/cm^3^), moderate decay time (150 ns), non-deliquescence, and non-self-irradiation. The scattering layer was an 8 × 8 HR-GAGG array of 2.5 mm × 2.5 mm × 1.5 mm scintillators that was used to detect Compton scattering of low-energy gamma rays from SPECT nuclides, and the absorbing layer was an 8 × 8 HR-GAGG array of 2.5 mm × 2.5 mm × 9 mm scintillators that was used to detect photon absorption of the 511 keV gamma rays from the PET nuclides. The scatterer thickness was decided so that a Compton scattering possibility would be more than 15% for an energy of 150 keV and less than 10% for an energy of 511 keV^22^. The pitch size was 3.2 mm, and each crystal was separated by BaSO_4_ reflectors. Each GAGG array was coupled to an 8 × 8 array of SiPMs (Hamamatsu MPPC S13361-3050) and was then wrapped with Teflon tape.

### Signal processing

The 64 channels of charge signals from the SiPMs were processed in parallel by using dynamic time-over-threshold (dToT) method^46,47^-based circuits. The ToT method^48–50^ offers several advantages—such as low power consumption and multi-channel parallel signal processing—over conventional pulse-height measurements with analog-to-digital converters (ADCs). Moreover, by using a dynamic threshold, the dToT method improves the non-linearity between the time width and the radiation energy, which is a disadvantage of the conventional ToT method. The dynamic threshold is determined by the rise and delay times. Each dToT-based circuit is comprised of an amplifier, a dynamic-threshold generator, and a comparator. The dynamic-threshold generator consists of two monostable multivibrators containing resistors and capacitors, which determine the rise and delay times. These parameters were optimized at 660 ns and 136 ns, respectively, by measuring the shapes of outputs from an amplifier for gamma rays with energies of 31 keV, 81 keV, 356 keV, 384 keV (from ^133^Ba), 59.54 keV (from ^241^Am), and 122 keV (from ^57^Co). The 64-channel dToT circuits are implemented on a board that enables parallel signal processing. The operating voltage is 3.3 V.

### Data-acquisition system

The 128-channel ToT outputs from a scatterer and an absorber are transferred to a 144-channel field-programmable gate array (FPGA, Xilinx Kinetex7 XC7k70T) data-acquisition (DAQ) system through KEL coaxial cables. The time stamp, channel number, and ToT pulse width are recorded in list mode in a solid-state drive (SSD) with 2.5 ns accuracy. As the prototype Compton-PET camera requires two DAQs, external clocks with a frequency of 1 kHz generated by a function generator are also transferred to the DAQs, utilizing an unused channel to synchronize the two DAQs. Time stamps are corrected using external clocks during offline analysis for extracting PET events.

### Analysis

As the nonlinearity between ToT width and energy is caused by the scintillators and SiPMs and dToT circuits, we measured the spectrum of several sources for calibration. The energy from the ToT response was calibrated by using the peak-position energies of several gamma rays: 31 keV, 81 keV, 356 keV, 384 keV (from ^133^Ba), 59.54 keV (from ^241^Am), 122 keV (from ^57^Co), 171 keV, 245 keV (from ^111^In), 511 keV (from ^22^Na), and 662 keV (from ^137^Cs).

**Figure 7:**
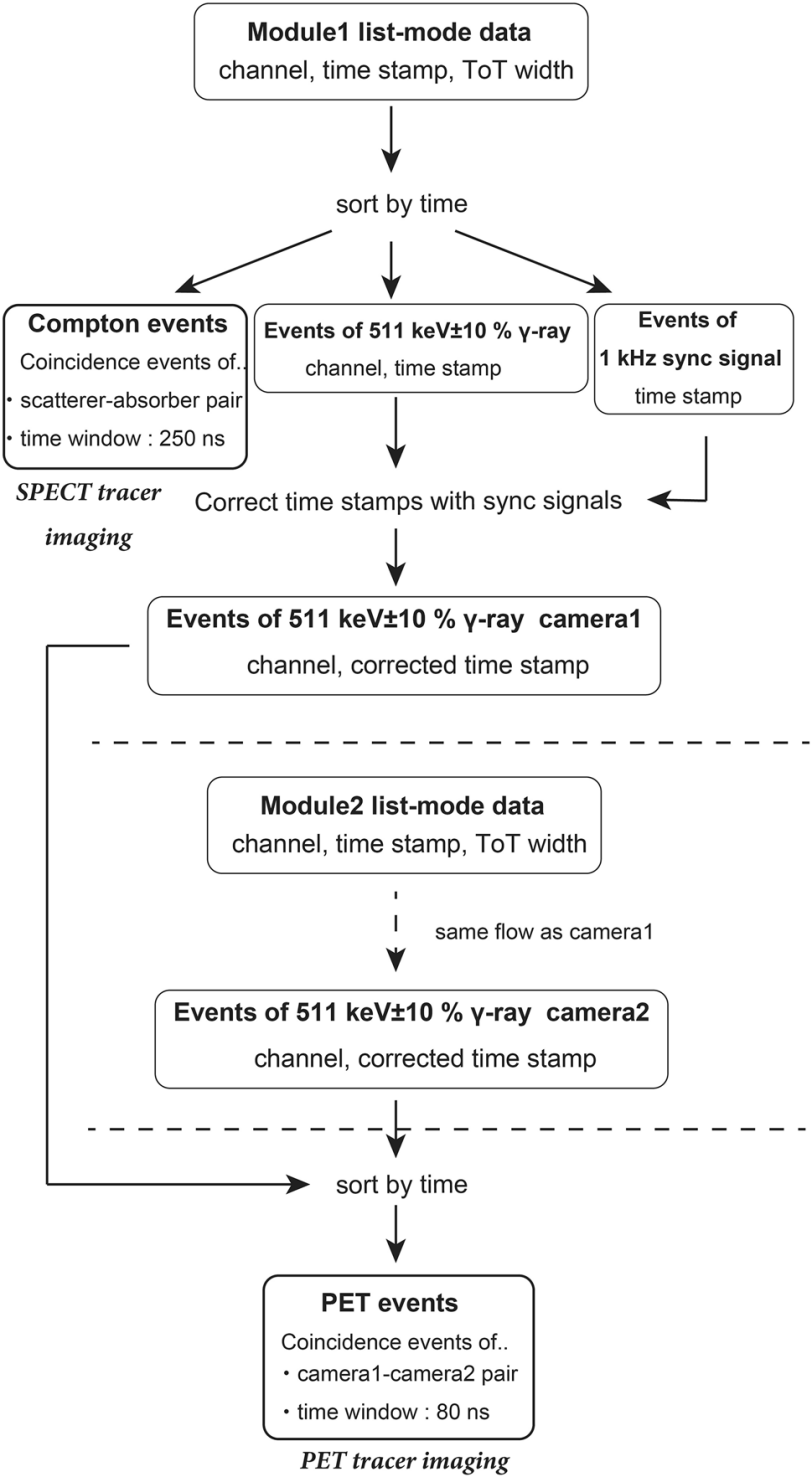
Analysis flow. List-mode data from each module contain information about the pixel channel, time stamp, and ToT width. First, all events from each module are sorted by time. Next, Compton events (events within 250 ns in a scatterer and an absorber), 511 keV gamma-ray-peak events, and sync-signal time stamps are extracted from each module’s data. Compton events are used for SPECT-tracer imaging. PET events (events within 80 ns in opposing absorbers) are extracted from the integrated photo peak events in the two modules after correcting the time stamps using the sync signals. PET events are used for PET-tracer imaging.

The data-analysis flow is shown in Fig. 7. Scatterer–absorber coincidence events within a time difference of 250 ns were selected from the list-mode data as Compton events after sorting by the time stamps. In addition, fully absorbed gamma-ray events within the energy range 511 keV $\pm \hspace{0.17em}$10% full width at half maximum (FWHM) and 1 kHz sync-signal events were extracted from the list-mode data. The time stamps of the 511 keV gamma-ray events were corrected by using the time stamps of the 1 kHz sync signal to adjust the time stamps of the data between the two cameras. The 511 keV gamma-ray events from the two cameras were integrated and sorted using the corrected time stamps. Then, coincidence events of the 511 keV annihilation gamma rays—within a time difference of 80 ns—were selected as PET events. The Compton events and PET events were used for Compton imaging and PET imaging to visualize the SPECT tracers and the PET tracers, respectively. The *x, y, z* coordinates of the positions of energy deposition were determined from the configuration of the detectors. In this paper, Compton images were reconstructed by using the MLEM method, and the PET images were reconstructed with the BP method.

### Experimental setup

**Figure 8:**
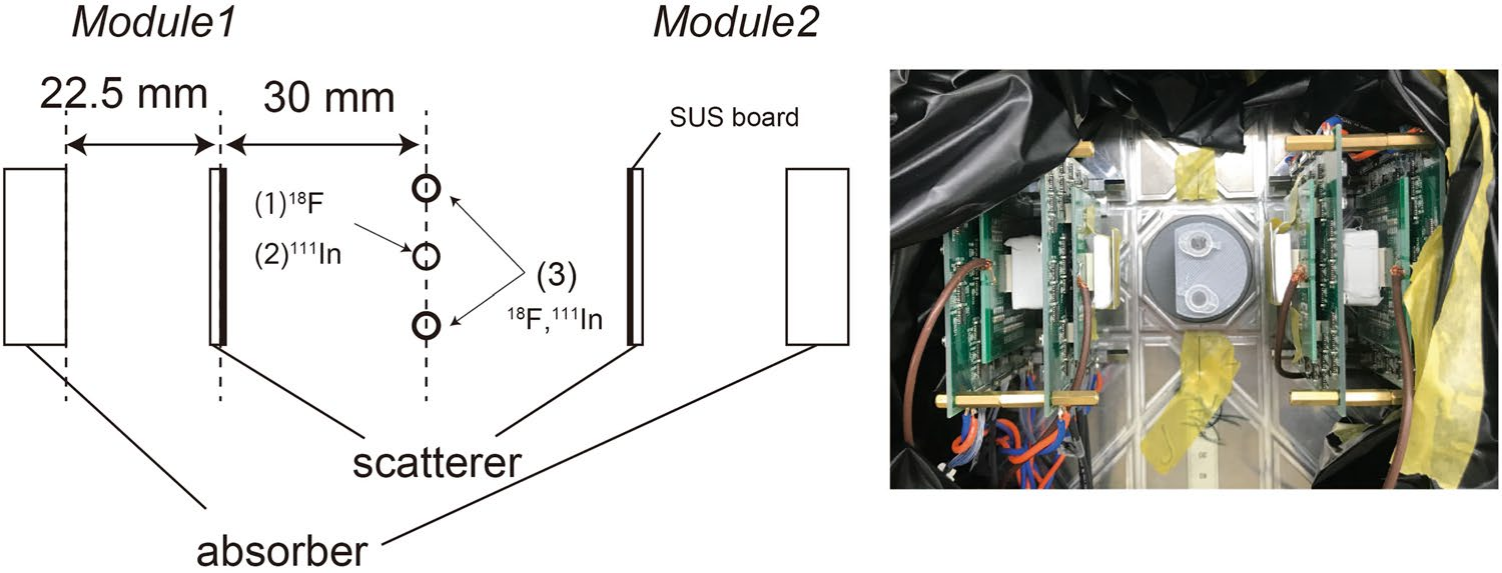
Experimental setup. Two scatterer–absorber modules were placed in opposing positions. The distance between the source and the surface of a scatterer was 30 mm, and the distance between the surface of a scatterer and that of an absorber was 22.5 mm. For the basic performance experiments, radionuclide sources were placed in the center between the two modules. SUS boards were placed in front of each scatterer to shield it from the ^111^In X-rays.

We evaluated the basic performance of (1) PET-nuclide imaging and (2) SPECT-nuclide imaging, and (3) we demonstrated simultaneous ^18^F-FDG (PET nuclide) and ^111^In (SPECT nuclide) imaging using micro tubes. For the simultaneous-imaging experiment, ^18^F-FDG was used as the PET nuclide, and ^111^In was used as the SPECT nuclide. Figure 8 shows the experimental setup with the prototype Compton-PET hybrid camera. Two scatterer–absorber modules were placed in opposing positions. The distance between the center and the scatterer surface was 30 mm, and the distance between the scatterer surface and the absorber surface was 22.5 mm. The distance between the scatterer and the absorber was determined to be as close as possible because the detection efficiency of higher energy Compton scattering events becomes worse depending on the distance. Although ARM becomes worse as the distance gets closer because of the geometry uncertainty, the detection efficiency of Compton scattering events of 245 keV gamma-ray was more priority in this experiment. The intrinsic detection efficiency of one Compton camera for 245 keV gamma-rays is approximately 0.039%. For experiments (2) and (3) using ^111^In, a 0.5-mm-thick steel use stainless (SUS) board was placed in front of each scatterer to eliminate the 22 keV X-rays from ^111^In.

### Evaluation of basic performance

The energy resolutions, spatial resolutions and time resolution (FWHM) were calculated from Gaussian fits. The ARM was evaluated as FWHM of the distribution of ${\theta }_{Ene}-{\theta }_{Geo}$, where ${\theta }_{Ene}$ is the angle calculated from Eq. (1), and ${\theta }_{Geo}$ is the angle defined by the geometric locations of the radioisotopes and the energy-deposition positions. The voigt function was used to determine the ARM. A ^22^Na point source was used for the evaluation of spatial resolutions of the PET image and time resolution. A ^18^F source and ^111^In source in 0.2 mL micro tubes were used for the evaluation of spatial resolutions of the Compton image and the ARM.

### Mouse preparation

We used a male nude mouse (BALB/c-nu/mu; 7 weeks old; JapanSLC) bearing an SY tumor, a small-cell lung-cancer cell line. This experiment was approved by the Committee of Care and Use of Laboratory Animals of National Institute of Radiological Sciences and was performed in accordance with the institutional guidelines and regulations. The experiment protocol complied with the ARRIVE guidelines. A tumor $\sim \hspace{0.17em}$10 mm in diameter was located on the left shoulder. We used ^18^F-FDG and ^111^InCl_3_ as the PET tracer and SPECT tracer, respectively. In this experiment, we injected 1.8 MBq of ^111^In-labeled antibody (67A2; anti-c-kit mouse monoclonal antibody) into the mouse approximately 3 days before the measurement. We also injected 0.2 MBq of ^18^F-FDG into the mouse 20 min before the measurement. The ^18^F radioactivity was lower than that of ^111^In in order to suppress artifacts in the images caused by crosstalk elements. The experimental setup of the two modules was the same as in Fig. 8, and the mouse was placed at the center between the two modules, and scanned under anesthesia (isoflurane inhalation of 1.0–1.5% in air).

## References

[1] Knoll GF (1983). Single-photon emission computed tomography. Proc. IEEE.

[2] Brownell GL (1958). Theory of radioisotope scanning. Int. J. Appl. Radiat. Isot..

[3] Ter-Pogossian MM (1975). A positron-emission transaxial tomograph for nuclear imaging (PETT) 1. Radiology.

[4] Kaida H (2010). Glucose transporter expression of an esophageal gastrointestinal tumor detected by F-18 FDG PET/CT. Clin. Nucl. Med..

[5] Adekola K (2012). Glucose transporters in cancer metabolism. Curr. Opin. Oncol..

[6] Iagaru A, Gambhir SS, Goris ML (2008). 90Y-ibritumomab therapy in refractory non-Hodgkin's lymphoma: Observations from 111In-ibritumomab pretreatment imaging. J. Nucl. Med..

[7] Delpassand ES (2008). Safety and efficacy of radionuclide therapy with high-activity In-111 pentetreotide in patients with progressive neuroendocrine tumors. Cancer.

[8] Caobellii F (2017). Simultaneous dual-isotope solid-state detector SPECT for improved tracking of white blood cells in suspected endocarditis. Eur. Heart J..

[9] Hijnen NM, de Vries A, Nicolay K, Grüll H (2012). Dual-isotope ^111^In/^177^Lu SPECT imaging as a tool in molecular imaging tracer design. Contrast Media Mol. Imaging.

[10] Wagenaar, D. J., et al. In vivo dual-isotope SPECT imaging with improved energy resolution. *2006 IEEE Nuclear Science Symposium Conference Record*. **6**, 3821–3826. IEEE. 10.1109/NSSMIC.2006.353824 (2006).

[11] Goorden MC (2013). VECTor: A preclinical imaging system for simultaneous submillimeter SPECT and PET. J. Nucl. Med..

[12] Todd RW, Nightingale JM, Everett DB (1974). A proposed γ camera. Nature.

[13] Watanabe S (2005). A Si/CdTe semiconductor Compton camera. IEEE Trans. Nucl. Sci..

[14] Takeda A (2011). Observation of diffuse cosmic and atmospheric gamma rays at balloon altitudes with an electron-tracking Compton camera. Astrophys. J..

[15] Sato Y (2018). Radiation imaging using a compact Compton camera inside the Fukushima Daiichi Nuclear Power Station building. J. Nucl. Sci. Technol..

[16] Lee W, Lee T (2010). A compact Compton camera using scintillators for the investigation of nuclear materials. NIMA.

[17] Jiang J (2016). A prototype of aerial radiation monitoring system using an unmanned helicopter mounting a GAGG scintillator Compton camera. J. Nucl. Sci. Technol..

[18] Motomura S (2008). Multiple molecular simultaneous imaging in a live mouse using semiconductor Compton camera. J. Anal. At. Spectrom..

[19] Kishimoto A (2017). First demonstration of multi-color 3-D in vivo imaging using ultra-compact Compton camera. Sci. Rep..

[20] Sakai M (2018). In vivo simultaneous imaging with 99mTc and 18F using a Compton camera. Phys. Med. Biol..

[21] Nakano T (2020). Imaging of 99mTc-DMSA and 18F-FDG in humans using a Si/CdTe Compton camera. Phys. Med. Biol..

[22] Shimazoe K (2020). Development of simultaneous PET and Compton imaging using GAGG-SiPM based pixel detectors. NIMA.

[23] Wilderman, S. J., et al. List-mode maximum likelihood reconstruction of Compton scatter camera images in nuclear medicine. *1998 IEEE Nuclear Science Symposium Conference Record. 1998 IEEE Nuclear Science Symposium and Medical Imaging Conference. (Cat. No. 98CH36255)*. **Vol. 3** IEEE, 1998. 10.1109/NSSMIC.1998.77387 (1998).

[24] Sakai M (2020). Crosstalk reduction using a dual energy window scatter correction in Compton imaging. Sensor.

[25] Yoshida C (2013). Therapeutic efficacy of c-kit-targeted radioimmunotherapy using 90Y-labeled anti-c-kit antibodies in a mouse model of small cell lung cancer. PLoS ONE.

[26] Tsuji AB (2012). Comparison of 2-amino-[3-11C] isobutyric acid and 2-deoxy-2-[18F] fluoro-D-glucose in nude mice with xenografted tumors and acute inflammation. Nucl. Med. Commun..

[27] Kato K (2011). An efficient and expedient method for the synthesis of 11C-labeled α-aminoisobutyric acid: A tumor imaging agent potentially useful for cancer diagnosis. Bioorg. Med. Chem. Lett..

[28] Visser EP (2009). Spatial resolution and sensitivity of the Inveon small-animal PET scanner. J. Nucl. Med..

[29] Wang Y (2006). Performance evaluation of the GE healthcare eXplore VISTA dual-ring small-animal PET scanner. J. Nucl. Med..

[30] Kishimoto A (2015). Demonstration of three-dimensional imaging based on handheld Compton camera. J. Instrum..

[31] Tashima H (2020). 3D Compton image reconstruction method for whole gamma imaging. Phys. Med. Biol..

[32] Yamaya T (2006). Preliminary resolution performance of the prototype system for a 4-layer DOI-PET scanner: JPET-D4. IEEE Trans. Nucl. Sci..

[33] Kishimoto A (2013). Development of a dual sided readout DOI-PET module using large-area monolithic MPPC-arrays. IEEE Trans. Nucl. Sci..

[34] Lee MS (2017). Prototype pre-clinical PET scanner with depth-of-interaction measurements using single-layer crystal array and single-ended readout. Phys. Med. Biol..

[35] Moses WW, Derenzo SE (1999). Prospects for time-of-flight PET using LSO scintillator. IEEE Trans. Nucl. Sci..

[36] Vandenberghe S (2016). Recent developments in time-of-flight PET. EJNMMI Phys..

[37] Hsu DFC (2017). Studies of a next-generation silicon-photomultiplier-based time-of-flight PET/CT system. J. Nucl. Med..

[38] Kim D, Uenomachi M, Shimazoe K, Takahashi H (2021). Evaluation of single scattering correction method in compton imaging system. NIMA.

[39] Shimzoe K (2017). Double photon emission coincidence imaging using GAGG-SiPM pixel detectors. J. Instrum..

[40] Uenomachi M (2020). Double photon emission coincidence imaging with GAGG-SiPM Compton camera. NIMA.

[41] Orita T (2021). Double-photon emission imaging with high-resolution Si/CdTe Compton cameras. IEEE Trans. Nucl. Sci..

[42] Uenomachi M, Shimazoe K, Ogane K, Takahashi H (2021). Simultaneous multi-nuclide imaging via double-photon coincidence method with parallel hole collimators. Sci. Rep..

[43] Sarkar SD (2002). Comparison of 123I and 131I for whole-body imaging in thyroid cancer. J. Nucl. Med..

[44] Garkavij M (2010). 177Lu-[DOTA0, Tyr3] octreotate therapy in patients with disseminated neuroendocrine tumors: Analysis of dosimetry with impact on future therapeutic strategy. Cancer.

[45] Kamada K (2014). Cz grown 2-in. size Ce:Gd_3_(Al, Ga)_5_O_12_ single crystal; relationship between Al, Ga site occupancy and scintillation properties. Opt. Mater..

[46] Shimazoe K (2012). Dynamic time over threshold method. IEEE Trans. Nucl. Sci..

[47] Orita T, Shimazoe K, Takahashi H (2015). The dynamic time-over-threshold method for multi-channel APD based gamma-ray detectors. NIMA.

[48] Kipnis I (1997). A time-over-threshold machine: The readout integrated circuit for the BABAR Silicon Vertex Tracker. IEEE Trans. Nucl. Sci..

[49] Powolny F (2008). A novel time-based readout scheme for a combined PET-CT detector using APDs. IEEE Trans. Nucl. Sci..

[50] Deng Z, Lan AK, Sun X, Bircher C, Liu Y, Shao Y (2011). Development of an eight-channel time-based readout ASIC for PET applications. IEEE Trans. Nucl. Sci..

